# On the Cryptogamous Origin of Malarious and Epidemic Fevers

**Published:** 1849-11

**Authors:** 


### ARTICLE IV.

*On the Cryptogamous Origin of Malarious and Epidemic Fevers.* By J. R. Mitchell, A.M., M.D.; Professor of Practical Medicine in the Jefferson Medical College of Philadelphia. pp. 137. octavo. Philadelphia: Lea & Blanchard. 1849. (From the publishers by S. C. Griggs & Co., Chicago.)

This is a doctrine of Fevers that appears to be original with Prof. Mitchell, and as a speculation we cannot see but that it is as good as any other. But it is theorizing that has put the great stumbling blocks in the way of the student of medical science, and in his attempts to pass around them he is continually led astray. We do not feel gratified with a perusal of the work, although it is very entertaining, and written in a style worthy of the high literary reputation of its distinguished author; for it lacks evidence sufficiently positive to give confidence in the truth of the doctrine, and we find we have only been following the author in his *gleanings for facts* to support his theory, many of which, it is true, are striking, but most of them are drawn from a great way off, and are often accidental expressions carelessly made. Should a man, occupying the position that the author does, to give weight and authority to his opinions, put forth a doubtful theory in such manner as, by argument, to convince his pupils, while he himself is not convinced? even though he warns them that he is making an *ex parte* use of the evidence he lays before them. Or is it consistent and proper teaching, for the professor, on so important a subject as fevers, to lay aside the didactic for the polemic style, and attempt to prove to his pupils what he scarcely believes to be true himself.

The little work before us is, as we learn by its dedication, the teaching of the Professor of Practical Medicine in Jefferson Medical College of Philadelphia, upon the subject of Fevers, to his class in the college, and is published at their request.

The book opens with very ingenious arguments against the various theories that have been advanced to explain the ætiology of malarious diseases,—one after another being disposed of for want evidence in its favor, until in speaking of the animalcular theory, his 4th and last argument is: "But the strongest objection is founded on the superior probability of the *vegetable branch of the organic theory*, &c."

In opening the argument in favor of his hypothsis, the author says:

"Not thorougly convinced myself, I can only be excused for occupying your time, by the belief that the theory I am about to offer, is not only very plausible, but is associated with agreeable and useful collateral inquiries. If we should not discover, at the end of our journey, the truth, the search after which has lured us to the path of observation, we shall enjoy, at least, beautiful scenery by the way; and sometimes pluck a flower, and sometimes find a gem."

Over twenty years ago the thought of the Cryptogamous origin of fevers was conceived by our author, on seeing some old stumps rapidly decay in a vicinity of much sickness. It led him to an investigation which resulted in the present development of a theory and its evidence. In reference to the latter, the author says: "You have, therefore, due notice of the guarded manner in which you are to receive my *ex parte* observations, a notice which I cheerfully give, for I have much confidence in the force of my subject, and do not love my theory well enough to wish its establishment at the expense of truth or reason."

The second lecture is principally occupied with a history of the fungi, which is continued nearly through the third. In this history some strong points of coincidence are shown between the habitudes of the fungi and the prevalence of several forms of disease.—As their greater growth in Autumn and at night, the time and season of the production and prevalence of malarious fevers.—Their more rapid growth in low situations and a certain degree of moisture, as well as the greater virulence of the poison of the fungi in damp weather, which conditions correspond with the prevalence of our fevers.

The remaining part of lecture 3d shows the frequency of mould, mildews, and other fungiferous productions, during epidemics. And the evidence of this is quite striking, forming in our estimation the strongest link in the argument; although much of it is questionable as to the true meaning of the observers. Instance:—"Plutarch, in his life of Romulus, says that in the first great plague at Rome, it semed '*to rain blood.*'" But the evidence seems to have very little reference to what we, in the West, regard as malarious diseases. Cholera,

Pestilence, Yellow Fever, Angina Maligna and Milzbrand, both of the latter being gangrenous diseases of cattle, and the Trembles, or Milk Sickness are especially cited.

But although fungi are exuberant during the prevalence of these epidemic and endemic affections in many instances, it does not appear that the affections are always to be found where the fungi are abundant. In fact, the proposition that the Plague or Cholera should be dependent upon a cause which we continually have among us, and often in great abundance, while they only appear once in a number of years: and the latter not at all until after the fungi had luxuriated for centuries among us, seems to be too wild a supposition to deserve serious argument. Nor is the supposition that it causes milk sickness more reasonable; for this disease is confined to limited neighborhoods of our country, while the supposed cause is universally to be found.

The 4th lecture is upon the poisonous qualities of the fungi, and the ravages they commit upon the health of man, beast and vegetables.

After the poisonous qualities are dwelt upon, the author observes:

"But it is rather to the peculiarities of these poisonings than the general fact that I would direct your attention. The first of these is the production of *fever*. Pereira tells us 'that the symptoms produced by the poisonous fungi are those of *gastro-intestinal irritation, and a disordered state of the nervous syetem*,' a not inexact general definition of a malarious fever."

Would any of our readers who observe fevers day after day and year after year, dream that gastro-intestinal irritation and a disordered state of the nervous system would be an exact general description of these diseases? True, it enumerates conditions that may attend these symptoms, but they are by no means uniformly present. To give an exact description requires that at least some diagnostic symptom should be cited.

If any one familiar with intermittent and remitting fevers, will look over the quotations from different authors to show the effects of mushroom poison, and from them make a good description of either of these fevers, we will award to him as much ingenuity as has been accredited to our author for the production of the work before us.

If mushroom poison be the cause of either intermittent, remitting, or congestive fever, how is it that persons when brought under the direct influence of the poison, do not have these diseases outright, instead of vague and unsatisfactory symptoms of them?

Our author says Christison relates a case of a woman and four children having a tertian fever after living four months exclusively on mushrooms, while the husband, who did not eat them, escaped. Two cases of intermittent fever, one in a lady of New York City, who had been eating mushrooms in the country, and the author himself, who had been handling and examining fungi, are referred to. This little evidence, at a point where the most is wanted, is made to suffice.

In the numerous cases related to bear upon the point under discussion, the evidence is stronger in favor of *gangrene*, than fever, resulting from fungi. Nearly all the cases cited mention the mortification. Does any of our readers recollect to have seen gangrene a frequent or general condition of malarious fevers?

Lecture 5th shows how the fungous theory explains the slow development of disease after malarious infection. This is done by citing that fungous poisoning takes effect at uncertain and various intervals after the poison is taken. From this simple fact our author concludes that the analogy is strong between the effects of this poison and malaria.

After this follows an explanation of the limitation of malarious diseases by, roads, walls and trees interposing. Here is a specimen of the argument:

"When we suppose that the poison is a fungous one, *progressively marching over the soil*, sustained by the rich air and pregnant moisture from the marsh, we can readily suppose that the wall, or the road, or the wood, may limit its progress. Besides this, the spores of all fungi are more or less electrical, and are, therefore, likely to be arrested by the trees of a wood.

"Authors have admitted that malaria appears to act in many instances as if it could exert no power, except when close to the spot where it originated, whilst in other cases, it seems to be wafted to a great distance from its apparent source. If we suppose the existence of germs susceptible of reproduction, and progressive growth, these seeming contradictions fall at once. The interruption of progress by a road or wall justifies this view of the mode of conveyance, and the many facts which show the narrow limits of the poisonous activity, enforce it strongly. The place, the very spot where the disease is found, must reproduce the cause of it for itself, and if the conditions of growth are not present, then will the spot be exempt, even if very near the most poisonous places. Thus may we, and only thus, explain the occurrence of agues, yellow fever, and cholera, on only one side of a house, or one end of a room, or one side of a street, or wall, or road."

Then is considered the effect of dried air. The wood choppers of Africa building fires near their work to advantage; Lind attributing the greater healthiness of one ship than another "to the location of her cooking apparatus between decks,"—Folchi, a Roman writer, saying that many persons, for years, in the worst parts of the country, live healthy by going in their houses before evening, closing up and warming the rooms, and remaining in doors until after sunrise—and old John Kaye's saying that smiths and cooks are free from the sweating sickness, form the evidence of the fact, and here is the conclusion:—"There is no other poison, save that of the fungi, so far as we know, which is thus disarmed by dryness and heat."

The cryptogamous nature of the fomites by which the yellow fever is often produced, is supposed, and a number of cases cited to prove that this disease may be carried in trunks and clothes. It is then assumed that there are but two ways of explaining the facts—contagion and fungi. And our author says as the disease is not contagious "there is left but one escape," fungi grow and propagate in the trunks.

Then the cholera is assumed to be a result of cryptogamous poisoning; and after stating some of the objections to contagion and animalculæ as causes, the author says:

"But if we assume for cholera a fungous origin, all difficulties vanish; and, as in the case of yellow fever, an easy explanation may be given of every apparent incongruity. We have only to suppose, what is known to happen in other cases, that the fungi, on which cholera is assumed to depend, acquire at times as do the germs of some contagious diseases, an unusual power of reproduction and diffusion, a greater potency of expansion. Such germs may be carried by men, and goods, and ships, or may make a slower progress by their own unaided activity, or be scattered by the winds, to regerminate, wherever special conditions are found. Thus can we see why the poison prefers the route of streams, or infests the damp parts of cities; and why classes living in clean apartments in dry districts, suffer so little.

"We can see why women escape better than men, why both cholera and yellow fever, by the natural tendency of the vegetable cause to the organs of generation, almost always cause miscarriage of pregnant women, and why, when a city or country is unhealthy, the fungiferous causes of death, by over-stimulating the organs of reproduction, usually make a compensation by the births, for the unusual mortality."

With as plastic a cause as this, could not any kind of disease be produced and explained? We have only to *suppose* at times an unusual power of reproduction, and such and such results *may* follow "wherever special conditions are found."

Who knows that fungi stimulate the organs of generation to greater activity in reproduction? and if no one, where the propriety of making an argument of the supposition? An article on the English Registrar General's Report, in the Medico Chirurgical Review says the number of births in England was greatly diminished during the prevalence of Cholera.

Our readers will most of them readily call to mind numerous cases of intermittent and remitting fever in pregnant women, without interfering with the function of gestation. In fact, intermittent fever by no means frequently produces abortion. The fungi are known to produce abortion even where they produce no other poisonous effect, as does ergot.

Do not all diseases of the violence of yellow fever and cholera, when attacking pregnant women, generally produce abortions? Most certainly.

The great difficulty in the way of explaining the cause of our malarious fevers, and perhaps of yellow fever, is that a cause cannot be found uniformly present where they prevail, and absent where they do not.

Our author cuts the knot in explaining the extraordinary exemption from fevers of certain localities, as Brazil, New Holland, and the Polynesian Islands, as follows:

"But if we assume the fungous theory as a basis of explanation, we may readily believe, nay, *certainly might know*, that such exceptions are, on the doctrine of chances, to be expected. No plant is everywhere, and such plants as are here alluded to, are especially capricious in habits and actions, according to causes which, though yet unstudied, obviously control them. On our theory, *the occasional exception should be looked for;* on any general chemical, or mechanical, or atmospheric theory, *it is inexplicable.*"

Certainly this is ingenious argument: but did it not occur to our author to ascertain whether the healthy localities were actually free from fungi, and the sickly ones uniformly attended by them?—To see if persons spending their time in damp mouldy rooms, where the country was generally healthy, would contract fever or not?

I know a man in this city who has been at work for a year in a small dark damp room where every thing around him has moulded in the course of a day or two at any time during the summer, leather especially became in a short time completely and densely covered, yet he has had no cholera, intermittent or remitting fever. I suppose his exemption can be accounted for "on the doctrine of chances."

Lecture 6th opens with a paragraph on the uncertainty of crops of all manner of vegetation, and shows that the variation is as yet unaccountable, and that the fungi are equally erratic in the manner of their production as shown by "the extension of only one disease or the co-existence of many," the author having assumed that each disease is dependent upon a distinct variety of the fungi. .

The sickness of the rainy season in Africa, is explained upon the theory, and it looks very plausible, taken in connexion with the asserted activity of mould, by which leather and clothes are rotted in a few hours. The fungiferous tendency of the volcanic *tufa*, our author thinks, explains the sickness of high and dry volcanic regions.—The porous stone favoring fungous growth so much as to be used for the cultivation of mushrooms for market. The immense consumption of mushrooms in Rome, averaging from 60,000 to 80,000 lbs., is cited to show the fungiferous tendencies of the country. The period of sickness following volcanic eruptions the succeeding year, as asserted by our author, is explained by the fungiferous tendency of the debris from the crater.

Again, the sickness of the seasons of great vegetation, and the exemption of others, is asserted to be the only approach of a cause, to constant coincidence with health and disease.—That the author is correct in this we have not the evidence to prove. The prevalence or non-prevlence of fevers in the West, we know does not corroborate the position, for the summer of 1848 was one of general abundance of crops, and of almost unprecedented health in the States of Indiana and Illinois.

The book closes with a recapitulation.

Taking it as a whole, the work presents manifest marks of a disposition to try the theory by a comparison with other doctrines, and to show it to be better than any other, instead of testing its truth or error. It is altogether plausible, that by the aid of "the doctrine of chances" the cryptogamous theory may be better than any other for the explanation of the course and character of fevers. But it is a poor apology for building an edifice upon the sand, that some one else built upon quick-sand. The Baconian doctrine has been so much harped upon, and it was almost universally agreed that medicine should be rid of speculation, and the system of drawing conclusions from facts alone be universally adopted, that we wonder at Prof. Mitchell for putting forth his theory until he had gone to the healthy localities, and to the sickly, in our own country, and there observed and tested the truth or error of his hypothesis, before he put it before the world, or taught it to medical students.

As the best addition; and one that almost completes the work, making it explain the causation of fever completely, we quote the following from Prof. Dickson on Malaria, in the N. York Jour. of Med. for Sept. '49:

"Now, if we suppose with Prof. (J. K.) Mitchell, an organized germ of vegetable character introduced, *qua data porta*, into the system, we may well imagine that its growth and multiplication will augment the quantity of deleterious matter into an efficient dose. Thus we account for the incubation of the attack. We further imagine then, either the death of of the first generation of fungi, or the elimination of the great mass of them through some of the emunctories, to explain the interval of intermission; and the birth and development of successive generations to render intelligible the long train of tenaciously recurring paroxysms. We imagine too that the several types of intermittent are owing to the variety of forms of cryptogamous vegetation, some of which propagate in one period and some in another."

Out upon suppositions, imaginations and guess work altogether. The profession has been cursed with them until it requires a nice amount of discrimination to determine what has been observed and what supposed, what is true and what guess work.

If it could be the happy fortune of our science, to have all the suppositions at once stricken from its records, the labor, error, and ignorance of a century would be avoided.

The student that studies suppositions and imaginations, acquires no knowledge that is worth the having, although he may "sometimes pluck a flower, and sometimes find a gem."

But it has become so much the custom to take the opinions and speculations of men eminent in the profession as our author deservedly is, without question or investigation, that they should be more than ordinarily guarded how they talk, lecture and write.

Nor is it a sufficient excuse for advancing "a theory not to be esteemed devoutly true," as the author says of this,* that it is less objectionable than others that are false. The more plausible, the more mischievious the doctrine, unless it be the true one. And even though there be a probability of the truth of a doctrine, this fact is far from justifying us in taking it as a foundation upon which to build a system, or an axiom from which to draw positive inferences. The proposition that there are more false facts than true ones, needs a little variation, and it will challenge contradiction. It is only the the substitution of *inferences* for facts. So long as investigations are entered into to prove preconceived opinions or theories, that long will we get wide of the truth, and grope our way in darkness.

*Page 39.

The work before us deserves the credit of great ingneuity, which has been awarded to it by most of our cotemporaries. First, it requires ingenuity to make six interesting lectures, or to write 137 octavo pages upon the fungous origin of fevers. Secondly, to collate the facts that bear upon the subject, either positively or apparently. Thirdly, to put the evidence together in such a way as to make the theory bear a plausible aspect. Fourthly, to make even an imperfect theory of malarious fevers in a region where these diseases are rarely to be seen or observed.

The doctrine that a man can investigate and understand, describe and illustrate a thing as well where it is not, as where it is, has been carried to a most ridiculous extent in reference to medical teaching, by those who assert "the gradually increasing feeling, that at least one session ought to be spent in Philadelphia. &c."* E.

*Announcement of Jefferson Medical College for 1849–50.

